# Global Trends in Research of Gouty Arthritis Over Past Decade: A Bibliometric Analysis

**DOI:** 10.3389/fimmu.2022.910400

**Published:** 2022-06-10

**Authors:** Pin Deng, Shulong Wang, Xiaojie Sun, Yinze Qi, Zhanhua Ma, Xuyue Pan, Huan Liang, Junde Wu, Zhaojun Chen

**Affiliations:** ^1^ School of Graduates, Beijing University of Chinese Medicine, Beijing, China; ^2^ Department of Hand and Foot Surgery, Beijing University of Chinese Medicine Third Affiliated Hospital, Beijing, China

**Keywords:** gouty arthritis, bibliometric study, VOSviewer, trends, CiteSpace

## Abstract

Gouty arthritis (GA), as a multifactorial disease, is characterised by intense pain, active inflammation symptoms, and swollen joints. It has utterly complex pathogenesis, of which the amount of research publications on GA has increased during the last few decades. A bibliometric analysis was carried out to investigate the trends, frontiers, and hot spots in global scientific output in GA research over the last decade. We retrieved the Science Citation Index Expanded (SCI-Expanded) of the Web of Science Core Collection (WoSCC) for publications and recorded information published from 2012 to 2021. we carried out the bibliometric analysis and visualisation analysis of the overall distribution of annual outputs, leading countries, active institutions, journals, authors, co-cited references, and keywords with the VOSviewer and CiteSpace. The impact and quality of papers were assessed using a global citation score (GCS). We retrieved 2052 articles and reviews in total. The annual number of publications (Np) related to GA research has increased during the latest decade. China published the most papers, and the USA achieved the highest H-index and number of citations (Nc). The League of European Research Universities (LERU) and Clinical Rheumatology (Clin Rheumatol) are the most productive institutions and periodicals. The total GCS of the paper written by Kottgen, A. in 2013 was 479, ranking the first. The most common keywords were “Gout,” “hyperuricemia,” and “gouty arthritis.” This research revealed that though there was a slight fluctuation in publications related to GA, the Np raised on the whole. China was an enormous creator, and the USA was an influential nation in this domain. The top three contributor authors were Dalbeth, N., Singh, JA., and Choi, HK. There were few investigations on the treatment of GA by Chinese medicine monomer, and the “mechanism,” “pathway”, “nf- kappa-b”, “injury”, “receptor”, and “animal model” were growing research hotspots. Our research illustrated the hotspots of research and development trends in the research field of GA during the last decade. Recognition of the most critical indicators (researchers, countries, institutes, and journals for the release of GA research), hotspots in the research field of GA can be helpful for countries, scholars, and policymakers in this field to understand GA better make decisions.

## Introduction

Gout is the most common type of inflammatory arthritis (1). Gouty arthritis (GA) is a metabolic disease involving the deposition of monosodium urate (MSU) crystals, a key initiator of acute inflammation (2). It is more common in postmenopausal obese women and middle-aged and older men. It generally manifests as a single afflicted joint with red, brilliant, and severe pressure pain, especially when the first metatarsophalangeal joint is implicated (3). GA affects around 2.1 million Americans, according to statistics from the National Health and Nutrition Examination Survey. Epidemiological evidence also indicates that the incidence of GA is rising dramatically worldwide (4). As a result, a quantitative analysis of the current situation, focus fields, and prospects for GA is critical.

Bibliometrics is an interdisciplinary discipline that has been used in gynaecology, orthopaedics, complementary and alternative medicine, and other medical domains. (5, 6). Based on evaluating databases and features of the literature, bibliometrics may estimate the developing trends in a scientific file and expose the research frontier as a handy technique. Furthermore, it can give reliable data that can be used to inform experimental tactics and financing decisions. (7). However, there is no bibliometric study on GA. Hence, this research aimed to investigate GA research in-depth to assess the current condition and hotspots in this domain.

## Methods

### Sources of Information and Search Methodologies

The literature dataset was compiled using Thomson-Web Reuter’s of Science Core Collection (WoSCC), which provides a comprehensive and standardised set of data for export and is widely used in academia. (8). The literature search was conducted in one day (19 April 2022) to avoid deviations, taking into account the rapid update of the database. The following were the search terms: (TS=(Arthritis, Gouty) OR TS=(Gouty Arthritis) OR TS=(“Gouty Arthritis”) OR TS=(“Gout Arthritis”) OR TS=(Arthritis, Gout) OR TS=(Gout Arthritis)**).** Among the various types of publications, Only English-language articles and reviews were considered. In total, 2052 articles were ultimately analysed from 2012 to 2021. The results of the thorough screening may be found in **Table 1**.

**Table 1:** TS search quires and refinement procedure.

Set	Results	Refinement
1	3297	TOPIC: (TS=(Arthritis, Gouty) OR TS=(Gouty Arthritis) OR TS=(“Gouty Arthritis”) OR TS=(“Gout Arthritis”) OR TS=(Arthritis, Gout) OR TS=(Gout Arthritis))) Indexes =SCI-EXPANDED
2	2415	Refined by PUBLICATION YEARS: (2012 OR 2013 OR 2014 OR 2015 OR 2016 OR 2017 OR 2018 OR 2019 OR 2020 OR 2021)
3	2107	Refined by DOCUMENT TYPES: (ARTICLES OR REVIEW ARTICLES)
4	2052	Refined by LANGUAGES: (ENGLISH)

### Data Collection

The data for all identified articles were obtained from the SCI-expanded database and then opened in Excel 2016, including authors, affiliations, countries/regions, journals, the number of papers and citations, publication year, H-index, keywords, and references. Two writers independently browsed the qualifying papers and extracted data. The data was then analysed further using an online program (http://www.bioinformatics.com.cn/), VOSviewer v1.6.10.0, and CiteSpace (version 5.8.R3).

### Bibliometric Analysis

The bibliometric analysis was carried out using VOSviewer (version 1.6.10), CiteSpace (version 5.8.R3), and the web tool (https://www.bibliometrix.org). The number of publications and citations often used to signify bibliographic material are examples of bibliometric indicators. As the two essential viewpoints for measuring research performance, the number of publications (Np) is often used to quantify productive capacity, and the number of citations (Nc) can demonstrate impact.

The H-index is primarily used to assess researchers’ academic contributions and forecast future scientific accomplishments. The H-index unites productivity and influence by identifying a threshold that links Np and Nc. A researcher will have an H-index if they have produced H articles, each quoted at least H times. Besides, The H-Index was designed to assess personal academic performance. However, it also may now define a country’s or region’s publishing output and an institution’s or journal’s production. (9, 10)

Additionally, the Impact Factor (IF) calculated from the most recent edition of the Journal Citation Reports (JCR) is widely recognised as one of the most important indices of medical journal quality and impact. (11). The Global Citation Score (GCS) is considered the Nc of an article worldwide. It is an essential indicator of the contribution an article makes to the field of knowledge, with a high GCS indicating a high level of interest from scientists worldwide (12). The fitted polynomial model was used to forecast the yearly Np further to demonstrate the variations in the annual publishing amount. The yearly number of investigations is represented by variable f (x), and the publication year is indicated by x.

VOSviewer v1.6.10.0 is used to construct and visualise bibliometric network graphs (Leiden University, Leiden, the Netherlands). (7, 13). VOSviewer was used to perform co-citation and co-occurrence analysis in this study. The size of the nodes represented the number of publications, the thickness of the line showed the strength of the relationship, and the colours of the nodes represented distinct clusters or periods.

Cluster analysis, timeline or time zone views, references, and keywords citation bursts were all employed in CiteSpace to aid in the visual evaluation of knowledge fields and developing trends. (14). Cluster analysis enables the categorisation of references and keywords, as well as the discovery of essential study topics for GA. Bursts of keywords and references are frequently utilised to discover new research trends.

## Results

### An Overview of GA Publications

A total of 2052 articles and reviews from the previous decade were retrieved from WoSCC, including 1,672 articles and 534 reviews. The total Nc of the retrieved articles was 32,131, and the average Nc per article was 19.29. The H-index for all publications was 79.

**Figure 1:**
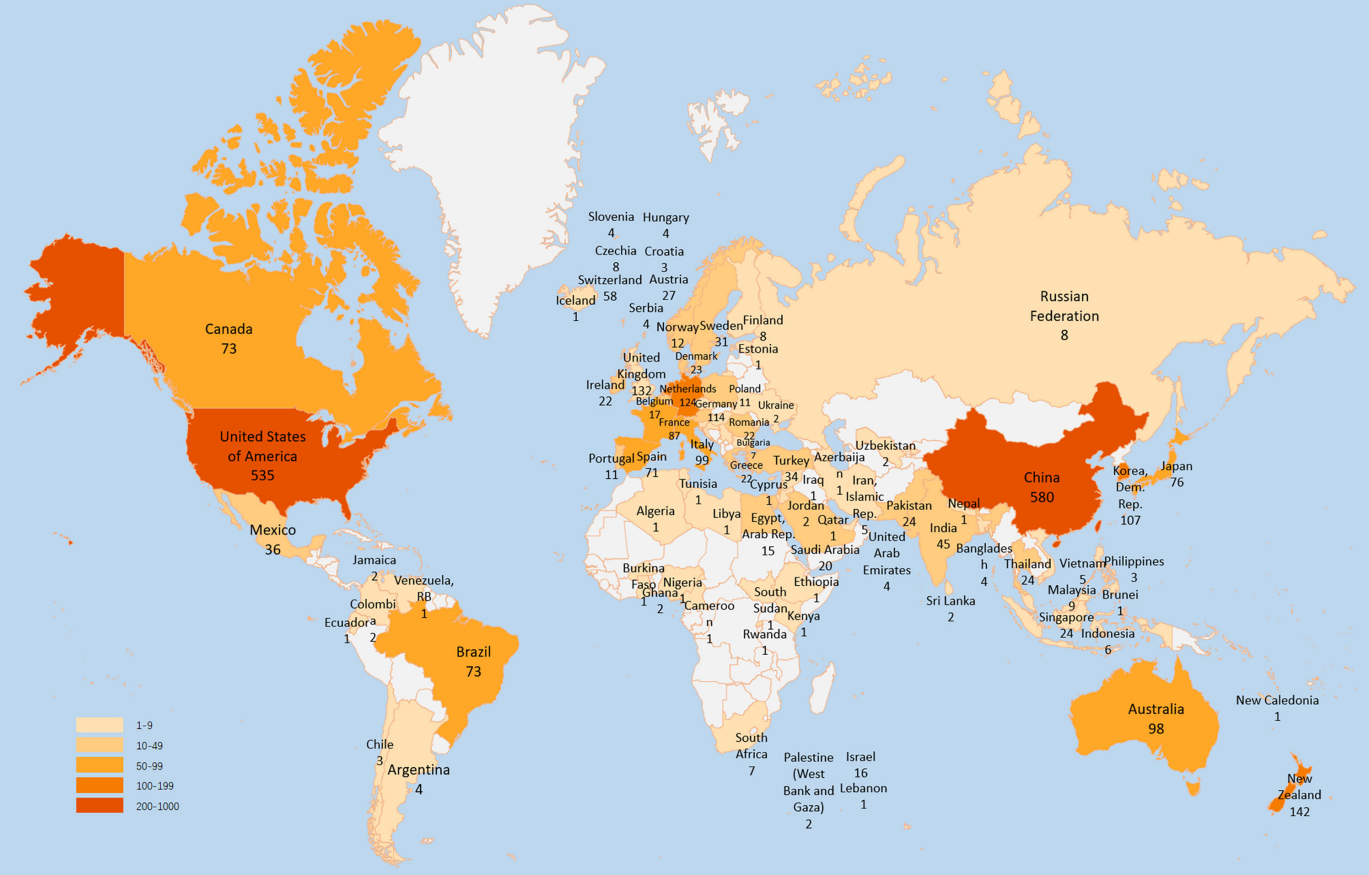
Geographical distribution of publications on GA research, 2012–2021.

**Figure 1** depicts the geographical distribution of the total number of papers on GA research from all nations and regions. The top five nations accounted for three-quarters of the 2052 articles. China was the country with the most papers published, followed by the USA, New Zealand, England, and Netherland.

**Figure 2:**
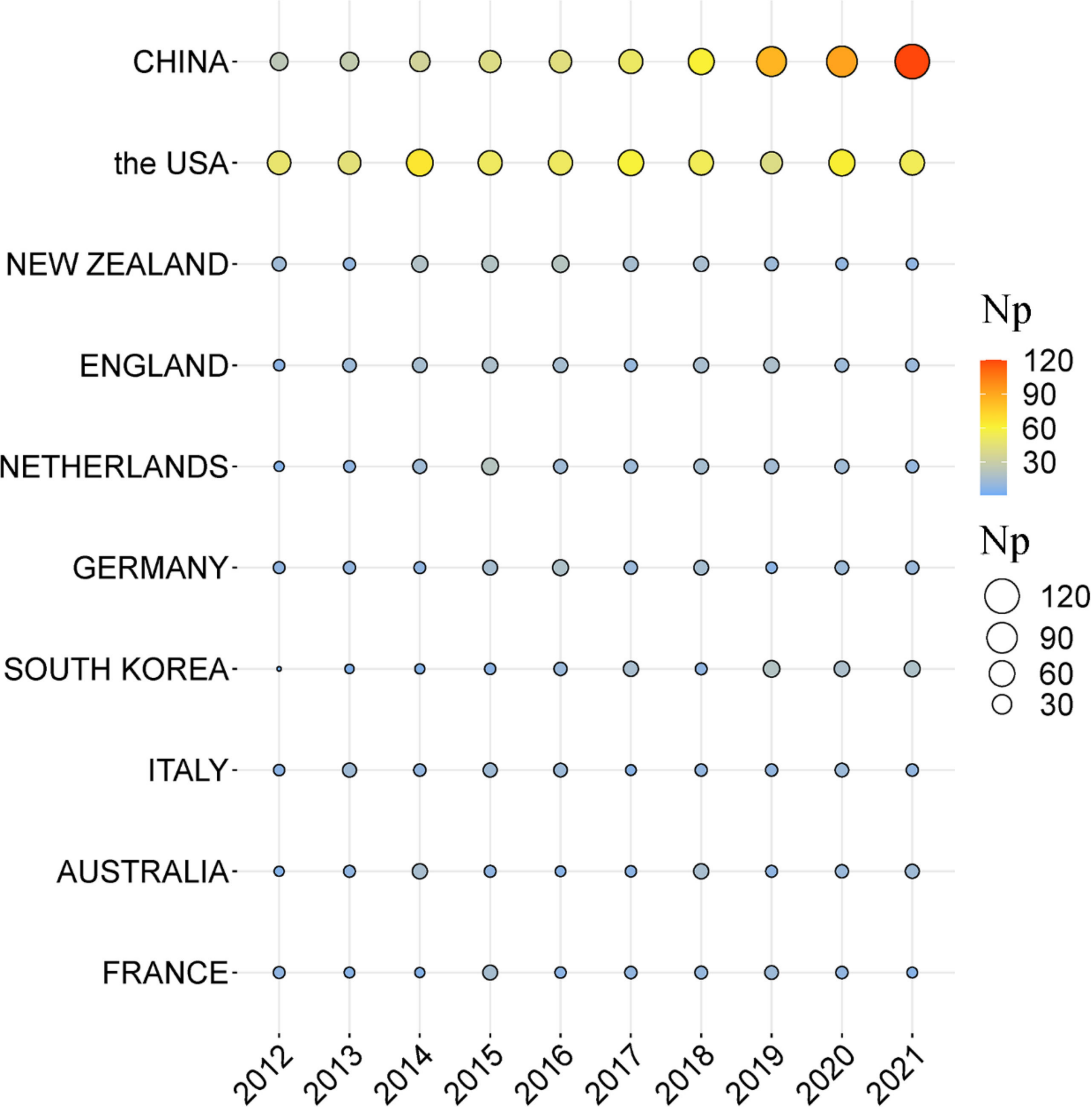
Top 10 countries in terms of annual publications on GA research, from 2012-2021. The circle’s size and colours show the number of papers. The larger the circle, the colour from light blue to red, the higher the number of articles issued in that country.

**Figure 2** describes the top ten countries in terms of annual publication on GA research from 2012-to 2021. China has the most significant number of articles published in 2021(120). Followed by the USA (54) and South Korea (18). It suggests that China remains the highest number of GA research.

### The Annual Trend in the Quantity of Paper Publication

**Figure 3:**
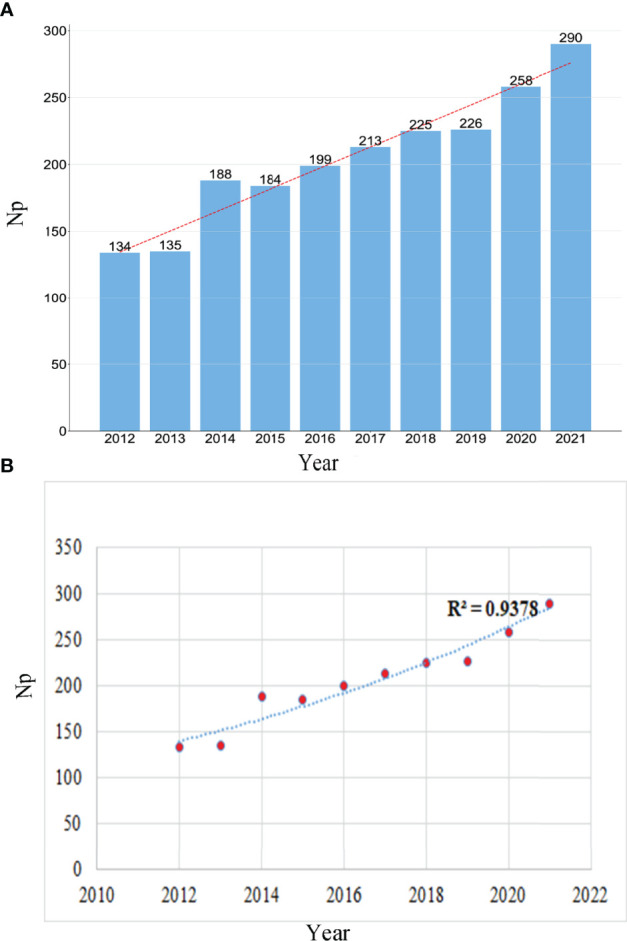
**(A)** The number of publications by year during the last ten years. **(B)** Curve fitting of publications’ overall yearly growth trend.

**Figure 3A** depicts the yearly Np as it relates to GA. The number of yearly papers increased from 134 in 2012 to 290 in 2021, and the Np peaked in 2021. China produces the most articles, indicating on the one hand that China is a high-yield country, and on the other hand, China pays more attention to GA research.

The polynomial-fitting curve of the yearly trend of paper publishing amount is depicted in **Figure 3B**. Despite minor fluctuations over ten years, there has been an overall tendency toward more articles being published, with the correlation coefficient R^2 =^ 0.9378, as shown in **Figure 3B**. Overall, these data imply that research on GA has been the centre of interest and has progressed rapidly.

### Countries’/Regions’ Contributions to Global Publications

We rated the top ten high-output countries/regions for all writers (**Table 2**). China published the most papers (580/28.26%), followed by the USA (535/26.07%) and New Zealand (142/6.92%). Papers from the USA were referenced 14607 times, accounting for 45.46 per cent of total citations, followed by papers from China (6422) and England (4189). Furthermore, the USA had the highest H-index (58), followed by China (38) and New Zealand (34). Compared to South Korea and Australia, France and Germany had a relatively lower Np but a much higher Nc and H-index. It may be related to APHP in France and LERU in Germany being in the top 10 institutions regarding the number of articles published. The two institutions play an essential role in expanding national influence.

**Table 2:** The top ten countries/regions with the highest productivity.

Rank	Country/Region	Np	% of (2052)	Nc	H-index
1	China	580	28.26%	6422	38
2	USA	535	26.07%	14607	58
3	New Zealand	142	6.92%	4116	34
4	England	132	6.43%	4189	32
5	Netherlands	124	6.04%	4062	32
6	Germany	114	5.56%	4177	32
7	South Korea	107	5.21%	1292	19
8	Italy	99	4.82%	2917	30
9	Australia	98	4.77%	3382	27
10	France	87	4.24%	4022	28

### Analysis of Affiliations

**Table 3** shows the top ten affiliations with the most GA-related publications. The highest Np was achieved by LERU (129). University of Auckland (101) is next, followed by the University of Otago (78). LERU had the highest Nc (4,824) and the greatest H-index (35). LERU is the University Alliance of Europe’s leading research universities and the principal advocate of promoting basic research in European universities. While Udice-French Research Universities and Assistance Publique Hopitaux Paris (APHP) in France have a little Np, they have a much higher NC than other productive colleges. Half of the affiliations were based in the USA. It suggests that more institutions in the USA are looking at gout arthritis research.

**Table 3:** The top ten most productive affiliations.

Rank	Affiliations	Country	Np	Nc	H-index
1	League of European Research Universities (LERU)	Germany	129	4824	35
2	University of Auckland	New Zealand	101	3467	31
3	University of Otago	New Zealand	78	2804	27
4	The Department of Veterans Affairs	USA	68	2728	24
5	Harvard University	USA	67	1669	23
6	Veterans Health Administration (VHA)	USA	65	2693	24
7	University of Alabama System	USA	59	2761	25
8	University of Alabama Birmingham	USA	58	2473	24
9	Udice-French Research Universities	France	56	3712	26
10	Assistance Publique Hopitaux Paris (APHP)	France	54	3215	26

### Analysis of Authors

**Table 4** lists the top ten most productive authors. They published 346 publications, accounting for 16.86 per cent of all articles submitted. Dalbeth, N (94). from the University of Auckland in New Zealand took first place in GA research, followed by Singh, JA (44). from Birmingham VA Med Ct and Choi, HK (34). from Harvard Med Sch in the USA. Dalbeth, N. had an extremely high Nc (3308). The work of Dalbeth N’s research has attracted more scholars’ attention. Furthermore, most of the top ten authors were mainly from the USA and New Zealand. It suggests that there are more excellent researchers in GA research in the US and New Zealand and focus on the field of GA research.

**Table 4:** The top ten authors with the most publications.

Rank	Author	Affiliations	Country	Np	Nc	H-index
1	Dalbeth, N.	Univ Auckland	New Zealand	94	3308	31
2	Singh, JA.	Birmingham VA Med Ct	USA	44	1804	17
3	Choi, HK.	Harvard Med Sch	USA	34	1110	20
4	Stamp, LK.	Univ Otago	New Zealand	34	863	18
5	Taylor, WJ.	Univ Otago	New Zealand	28	1341	17
6	Rome, K.	AUT Univ	New Zealand	25	238	10
7	Schlesinger, N.	Univ Med & Dent New Jersey	USA	25	504	12
8	Neogi, T.	Boston Univ	USA	24	1840	16
9	Bardin, T.	Assistance Publique Hopitaux Paris (APHP)	France	19	1276	14
10	Li, CG.	Qingdao Univ	China	19	185	6

### Analysis of Journals

As shown in **Table 5**, Clinical Rheumatology (Clin Rheumatol) (72 publications, IF: 2.98) published the most studies on GA. Clin Rheumatol aims to cover all current clinical and experimental research trends involving inflammatory, metabolic, immunologic, and degenerative soft and hard connective tissue diseases. Followed by Rheumatology (61 publications, IF:7.58) and Arthritis Research and Therapy (Arthritis Res Ther) (59 publications, IF:5.156). Approximately 24% of the publications were published in the top ten academic publications (483/23.53%). Except for Clin Rheumatol (IF:2.980) and Rheumatology International (IF:2.631), all of the top ten journals had a higher IF (defined as >3.000). It suggests that Clin Rheumatol and Rheumatology International should increase the influence of journals and the quality of articles published in the future while maintaining the number of articles published.

**Table 5:** The top ten most-published journals.

Rank	Journal	Np	Nc	H-index	IF(2020)
1	Clinical Rheumatology	72	801	19	2.98
2	Rheumatology	61	1531	24	7.58
3	Arthritis Research and Therapy	59	1540	24	5.156
4	Annals of the Rheumatic Diseases	51	2914	32	19.103
5	Journal of Rheumatology	50	852	18	4.666
6	Seminars in Arthritis and Rheumatism	46	1294	21	5.532
7	Rheumatology International	41	585	14	2.631
8	Arthritis Care and Research	35	2473	24	4.794
9	Journal of Ethnopharmacology	35	431	13	4.36
10	Joint Bone Spine	33	471	14	4.929

**Table 6** describes the number of publications of GA in the top Journals in Rheumatology (Arthritis and Rheumatology, Annals of the Rheumatic Diseases, Nature Reviews Rheumatology) and other journals with the high impact factors. The total Np of Nature Reviews Rheumatology (IF:20.543) is 13. The total Np of Arthritis & Rheumatology (IF:10.995) is 27, and the total Np of Annals of the Rheumatic Diseases (ARD) (19.103) is 51. Although the top journal has a relatively small number of articles on GA, However, the ARD is ranked 4th in the top ten journals in terms of the number of articles, suggesting that the research on GA has attracted some attention. The USA has the most articles in this journal, demonstrating that the USA values this journal and has an excellent scientific research base and scientific research strength.

**Table 6:** Correlation of high-quality journals with high attention and the most productive countries.

Rank	Journal	Total	Np			IF (2020)
			China	USA	New Zealand	
1	Rheumatology	61	9	14	6	7.58
2	Annals of the Rheumatic Diseases	51	5	26	16	19.103
3	Seminars in Arthritis and Rheumatism	46	2	29	6	5.532
4	Arthritis & Rheumatology	27	/	19	9	10.995
5	Nature Reviews Rheumatology	13	1	4	2	20.543

“/” represent no Np.

### Analysis of Highly Cited Articles

The articles in **Table 7** were listed in descending order of the total number of citations. The top ten most cited articles mainly were published between 2012 and 2016. Three articles have been cited more than 450 times, published in the Nature Genetics (IF= 38.33. title: Genome-wide association analyses identify 18 new loci associated with serum urate concentrations. type of study: Clinical Research, 491 citations). This article emphasises the significance of metabolic control of urate production and excretion, which may have implications for gout treatment and prevention. Follow by Arthritis Care &Research (IF=4.794, title: 2012 American College of Rheumatology guidelines for management of gout. Part 2: Therapy and antiinflammatory prophylaxis of acute gouty arthritis. Type of study: article, 484 citations), and Nature Medicine (IF=53.44. title: Aggregated neutrophil extracellular traps limit inflammation by degrading cytokines and chemokines. type of study: Basic and Clinical Research, 478 citations). Nature Medicine covers research that addresses the needs and goals of contemporary medicine. Original research ranges from new concepts in human biology and disease pathogenesis to robust preclinical bases for new therapeutic modalities and drug development. Studies related to GA were published in Nature Medicine, indicating that researchers attach great importance to the study of the pathogenesis of GA.

**Table 7:** The top ten highest cited articles.

Rank	Year	Article	IF (2020)	Total citation	Type of study
1	2013	Kottgen, A. et al. Genome-wide association analyses identify 18 new loci associated with serum urate concentrations. NATURE GENETICS. 2013, 45 (2): 145-154	38.33	491	Clinical Research
2	2012	Khanna, D. et al., 2012 American College of Rheumatology guidelines for management of gout. Part 2: Therapy and antiinflammatory prophylaxis of acute gouty arthritis. ARTHRITIS CARE & RESEARCH. 2012, 64 (10): 1447-1461	4.794	484	Article
3	2012	Schauer, C. et al. Aggregated neutrophil extracellular traps limit inflammation by degrading cytokines and chemokines. NATURE MEDICINE,2014, 20 (5): 511-517	53.44	478	Basic and Clinical Research
4	2012	Juliana, C. et al. Non-transcriptional Priming and Deubiquitination Regulate NLRP3 Inflammasome Activation. JOURNAL OF BIOLOGICAL CHEMISTRY. 2012, 287 (43): 36617-36622	5.157	446	Basic Medical Research
5	2013	Dinarello, CA. et al. Treating inflammation by blocking interleukin-1 in humans. SEMINARS IN IMMUNOLOGY. 2013, 25 (6): 469-484	11.13	321	Review
6	2015	Leung, YY. et al. Colchicine-Update on mechanisms of action and therapeutic uses. SEMINARS IN ARTHRITIS AND RHEUMATISM. 2015, 45 (3): 341-350	5.532	314	Review
7	2013	Graham, GG. et al. The modern pharmacology of paracetamol: therapeutic actions, mechanism of action, metabolism, toxicity and recent pharmacological findings. INFLAMMOPHARMACOLOGY. 2013, 21 (3): 201-232	4.473	294	Review
8	2015	Neogi, T. et al., 2015 Gout Classification Criteria An American College of Rheumatology/European League Against Rheumatism Collaborative Initiative. ARTHRITIS & RHEUMATOLOGY. 2015, 67 (10):2557-2568	10.995	291	Article
9	2014	March, L. et al. Burden of disability due to musculoskeletal (MSK) disorders. BEST PRACTICE & RESEARCH IN CLINICAL RHEUMATOLOGY. 2014, 28 (3): 353-366	4.098	287	Article
10	2016	Pei, KH. et al. p-Coumaric acid and its conjugates: dietary sources, pharmacokinetic properties and biological activities. JOURNAL OF THE SCIENCE OF FOOD AND AGRICULTURE. 2016, 96 (9): 2952-2962	3.639	233	Review

### Analysis of Paper Global Citation Score (GCS)

**Figure 4:**
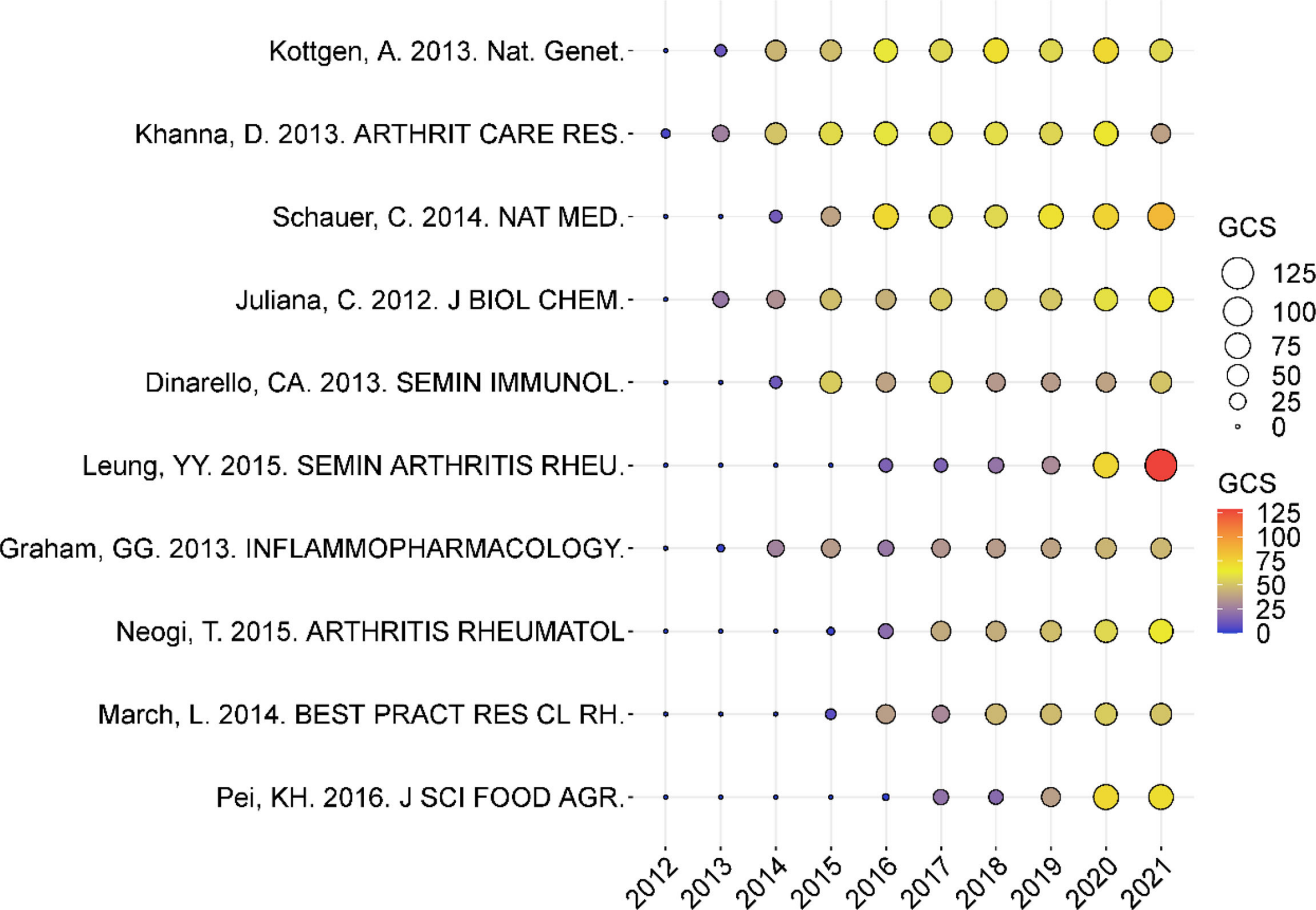
The yearly number of global citations for papers having a high global citation score (GCS). The circle’s size and colours show the GCS of papers. The larger the circle and the colour from blue to red, the higher the GCS of the article and the more influential it is in the field.

**Figure 5:**
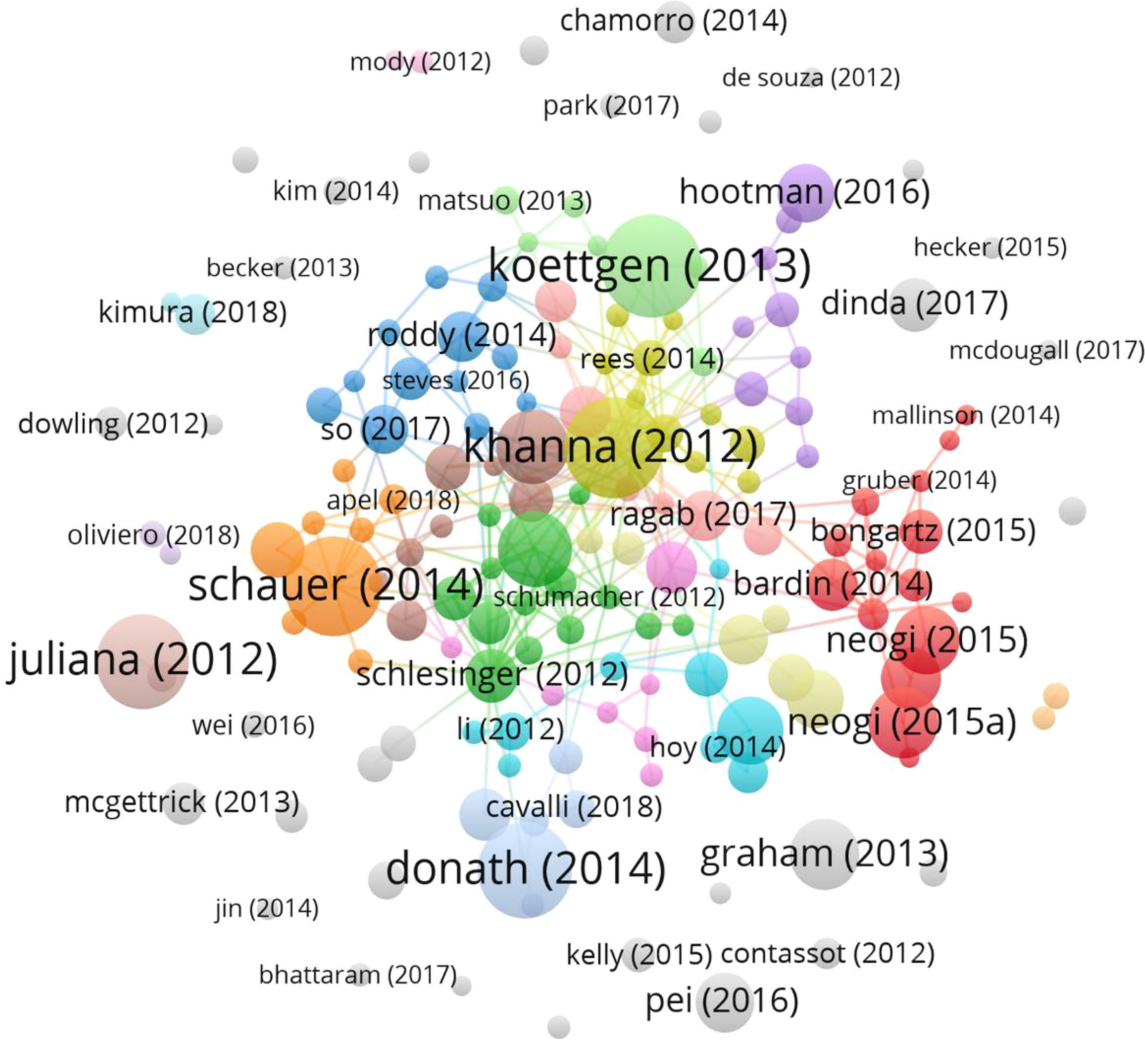
Network of document citation. Given a large number of references available, the minimum number of citations for a reference was placed at 50. 161 of the 2052 papers were chosen for citation analysis. The different colours of the nodes represent different documents, with larger nodes meaning more frequently cited articles.

**Figure 4** depicts the yearly number of global citations for papers having a high global citation score (GCS). The GCS of the paper written by Leung, YY. in 2012 was 128, ranking the first. In this paper, the authors referred to Colchicine’s newly described mechanism of action includes various inhibitory effects on macrophages, including inhibition of the NACHT-LRRPYD-containing protein 3 (NALP3) inflammasome. Colchicine’s therapeutic use has expanded beyond GA to include osteoarthritis, pericarditis, and atherosclerosis. (15). Followed by Schauer C. et al.’ research. His research found that aggregated NETs promote neutrophilic inflammation resolution by degrading cytokines and chemokines and disrupting neutrophil recruitment and activation. Additionally, the works of Juliana, C. et al. (16) about that NLRP3 inflammasome is involved in the pathogenesis of GA and that inhibiting NLRP3 activation has both preventive and therapeutic effects on GA have accumulated more GCS in recent years. The study of Pei, KH. et al. detailed P-Coumaric acid (P-CA) has analgesic, antipyretic, hypopigmented, anti-ulcer, anti-arthritis, antiplatelet aggregation, and anxiolytic activities. It has the potential to be utilised to treat GA. (17). Graham, GG. et al. pointed out that although paracetamol does not suppress the severe inflammation of rheumatoid arthritis and acute gout, it does reduce the less severe inflammation caused by tooth extraction and is active in a range of inflammatory tests in experimental animals. Furthermore, in Neogi T’s study, the authors thoroughly reviewed the advanced imaging literature on gout through the European League Against Rheumatism. They developed new classification criteria for gout, which increased the number of subsequent publications in the field. (18). March, L. et al. (19). summarised the worldwide and regional prevalence, disability, total burden, and costs of major musculoskeletal ailments such as knee osteoarthritis, rheumatoid arthritis, gout, etc. Taken as a whole, these documents have a significant effect on the study of GA, increasing the number of following papers on the subject. Most of the above studies focus on the mechanism and clinical treatment of GA. **Figure 5** shows the 161 articles (other publications are cited more than 50 times) with high citation frequency, corresponding to **Figure 4**, where the nodes of articles with high GCS are relatively large and are at the core of the mesh.

**Figure 6:**
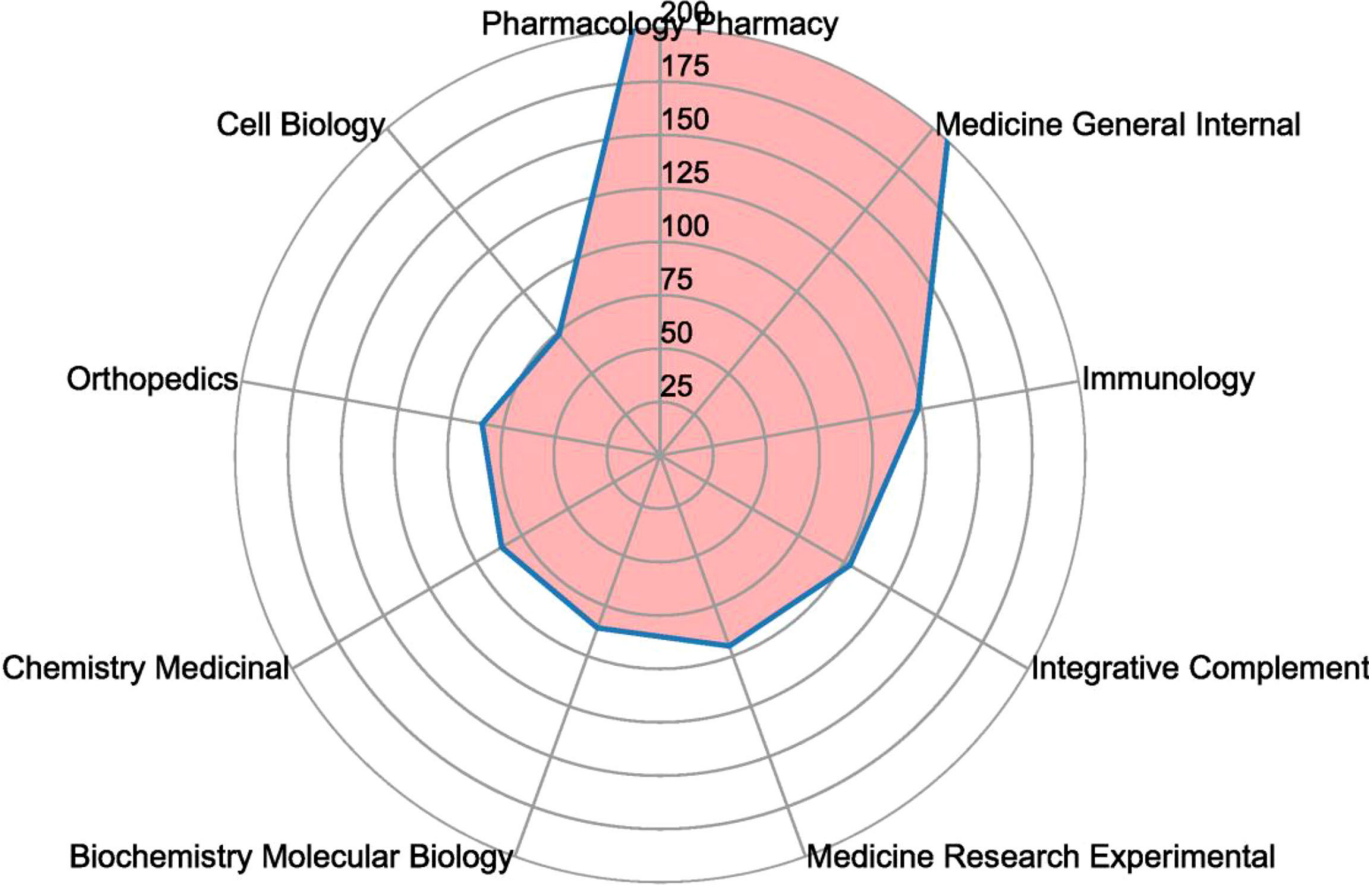
Radar map of the top ten research productive categories on GA.

**Figure 6** depicts the top ten productive categories associated with GA. The most prevalent study categories were Pharmacology Pharmacy (252 papers), followed by Medicine General Internal (213 papers), Immunology (123 papers), Integrative Complementary Medicine (103 papers), and Medicine Research Experimental (95 papers). Pharmacology Pharmacy had the highest proportion of publications in terms of research categories, while Cell Biology had the lowest proportion of publications.

### Analysis of Co-Cited References

**Figure 7:**
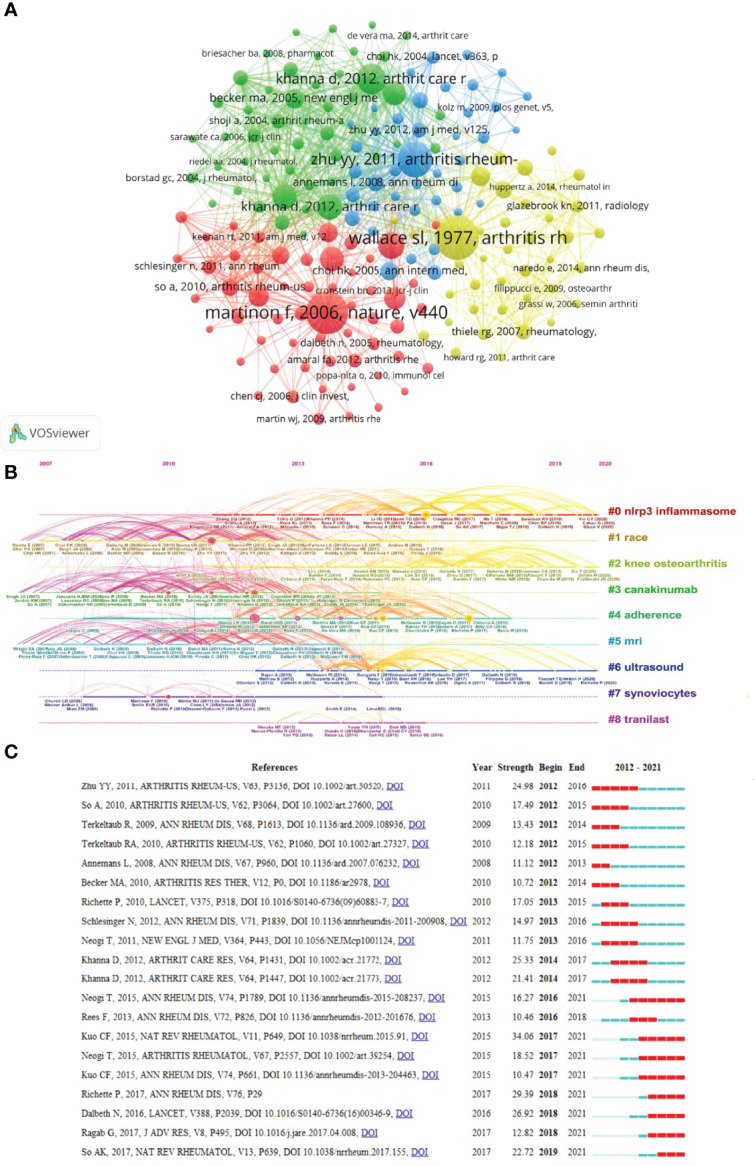
Mapping based on co-cited references from GA-related research. **(A)** A network diagram of co-cited references. Of the 51775 references, 184 (divided into four clusters) were cited at least 30 times. **(B)** The top nine clusters’ timeline distribution. **(C)** The top 20 co-cited references with the most citation burstiness. The years between “Begin” and “End” represent the period when the reference was more influential. Years in light green mean that the reference has not yet appeared, years in dark green mean that the reference is less influential, and years in red mean that the reference is more influential.

Co-citation networks emphasise research topics closely related to specific fields, different from global citation analysis. Given the enormous number of referenced references, a document’s minimum number of citations was 30. One hundred eighty-four references were chosen for co-citation analysis out of the 51775 mentioned by the retrieved publications. A line connecting two nodes indicates that they were referenced in the same publication, and a shorter line indicates a closer association between two publications. Furthermore, different node colours were employed to split the papers into clusters (**Figure 7A**). Cluster 1 (red) comprises 56 references, focusing on epidemiology, pathogenesis, and gout treatment. Cluster 2 (green) focused primarily on the clinical study of gout therapeutic drugs. Cluster 3 (blue) mainly focused on the prevalence, comorbidities and management of gout. The theme of cluster 4 (yellow) centred on the application of modern detection technology to diagnose gout. Based on the clustering, we discovered that most studies focused on the epidemiology and pathogenesis of gout. **Figure 7B** illustrates the most typical references in terms of burst length, burst strength, and burst time. As shown in **Figure 7B**, the top nine clusters of co-cited references were “nlrp3 inflammasome,” “race,” “knee osteoarthritis,” “canakinumab,” “adherence,” “mri,” “ultrasound,” “synovlocytes,” “tranilast.” **Figure 7C** depicts the top 20 references with the most powerful citation bursts. The Kuo CF et al. study has the highest strength (34.06). His article describes developments in the epidemiology of gout and patterns of urate-lowering therapy (ULT) in the UK general population. We also discovered that the studies of Richette, p. et al. (20) possess the higher bursts strength (29.39). In his paper, he revised the evidence-based gout management recommendations of the European League Against Rheumatism (EULAR). These recommendations are intended to educate physicians and patients about non-pharmacological and pharmacological gout therapies and the best tactics for achieving the predetermined urate target and curing the condition. In addition, So, Ak’s essay has seen a significant increase in citations. Recent developments in our understanding of positive and negative regulatory mechanisms and genetic and environmental variables that influence inflammatory responses, some of these mechanisms can be regulated, opening up new therapeutic possibilities for GA, are highlighted in his paper. (21). The above-mentioned high-strength literature has conducted relevant research on GA from a variety of perspectives, including epidemiological studies of GA, gout management recommendations, and research on the inflammatory response mechanism of GA.

### Analysis of Hotspots in Research

**Figure 8:**
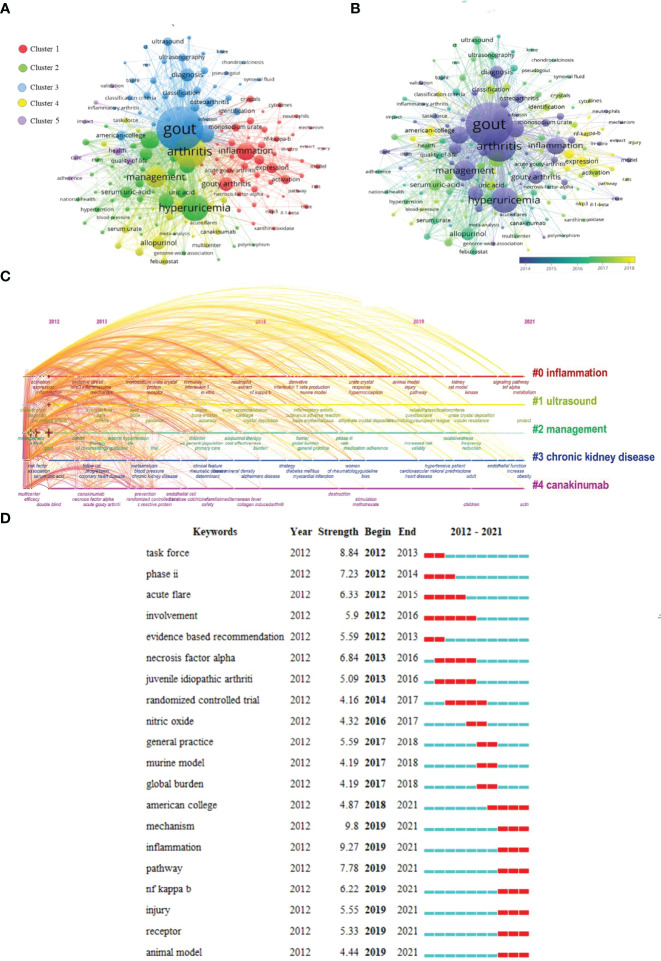
GA’s keyword mapping. **(A)** Using different colours, the 168 terms that appeared more than 20 times were separated into five clusters: Cluster 1 (red): the inflammatory mechanism of GA. Cluster 2 (green): the prognosis, and epidemiology of GA. Cluster 3 (blue): the research on the diagnosis of GA. Cluster 4 (yellow): the clinical research of GA. Cluster 5 (purple): the care and risk factors for GA. The size of the nodes indicates occurrence frequency. **(B)** Keyword visualization according to the APY. The different colours indicate the relevant year of publication. Yellow keywords came later than purple keywords. **(C)** Timeline distribution of keyword cluster analysis. **(D)** The top twenty keywords with the most bursts. The years between “Begin” and “End” represent the period when the keyword was more influential. Years in light green mean that the keyword has not yet appeared, years in dark green mean that the keyword is less influential, and years in red mean that the keyword is more influential.

Apart from search terms, VOSviewer and Citespace evaluated keywords collected from the titles and abstracts of 2052 publications. Clusters 1 (Red) primarily represented the inflammatory response mechanism to GA, as seen in **Figure 8A**. Cluster 2(Green) is primarily concerned with GA’s prognosis and epidemiology. Cluster 3 (blue) focused on the research on the diagnosis of GA. Cluster 4 (yellow) was mainly about the clinical research of GA. Cluster 5 (Purple) primarily focused on GA’s care and risk factors. The top frequent occurrences of keywords were “gout,” “arthritis,” “hyperuricemia,” “management,” and “inflammation,” suggesting that the research related to GA mainly focused on causes and mechanisms.

As seen in **Figure 8B**, VOSviewer separated the colours of all keywords into categories based on their average publication year (APY). The latest keyword was “injury” (cluster 1, APY:2019.50), followed by “nlrp3” (cluster 1, APY: 2018.95) and “pathway” (cluster 1, APY: 2018.88), both closely associated with GA. Besides, “nlrp3 inflammasome” (cluster 1, APY:2018.31) and “nf-kappa-b” (cluster 1, APY: 2018.25), as well as “expression” (cluster 1, APY: 2018. 03), were the field’s most recent hot topics. Additionally, “inflammation,” “ultrasound,” “management,” “chronic kidney disease,” and “Canakinumab” have long been a focus of research in GA, as shown in **Figure 8C**. Meanwhile, we discovered that the terms “pathway,” “nf kappa b,” “injury,” “receptor,” and “animal model” were the most recently trending keywords during the previous three years in **Figure 8D**.

The common thread in **Figures 8A–D** is research into the inflammatory response mechanism to GA. There will be more and more literature on the basic research of GA in the future, and more scholars will be inclined to explore the pathogenesis and treatment mechanism of GA using animal experiments. Then explore more possibilities for the treatment of GA.

## Discussion

This study is the first bibliometric study of global research on GA. This study aimed to undertake a bibliometric analysis of trends and research hotspots in GA utilising the SCI extended of the WoSCC database, VOSviewer, and Citespace. We retrieved 2052 articles and reviews published from 2012 to 2021. Although the number of papers published has a minor fluctuation during the decade, there has been an overall trend toward more articles being published, according to the polynomial fit curve. It also suggests that more researchers are becoming interested in the topic of GA.

Publications are dispersed worldwide, yet productivity in many locations is modest. **Figure 1** depicts the geographic distribution of publications on GA research. China has the most publications (n=580), followed by the USA (n=535), New Zealand (142) and England (n=132). China rated first in Np among the top ten countries/regions, indicating a very prolific country on GA. Five institutions and four scholars of the USA were in the top ten affiliations and authors in GA research, indicating that the USA has the most exceptional institutions and specialist scholars, which helped explain why the USA has had so much influence on this subject over the last decade.

Moreover, the USA has a comparatively high H-index and Nc compared to China. It is owing to American researchers first suggesting the recommendations for gout care and medication and antiinflammatory prophylaxis for AGA. (22) Furthermore, the USA was subjected to more intensive research on the topic than the rest of the globe. It is suggested that Chinese academics and affiliates in this subject should improve the quality of their works. Similarly, in South Korea, there is a disparity in the amount and quality of publications.

As far as affiliations are concerned. Almost all of the top ten institutions were from the top three countries with the most publications, half of which were in the USA, demonstrating the country’s good scholarly competence in this field. Dalbeth, N, Singh, JA, and Choi, HK were the top three scholars in the research of GA with the most publications. As a result, to keep up with the latest developments in this domain, we should place a greater emphasis on their work and give it a higher priority. LERU (published 129 articles, cited 4824 times) is the most productive institution. Followed by the University of Auckland (published 101 articles, cited 3467 times), and Dalbeth produced the majority of the papers, N’s team from this institution has a long-term research focus dedicated to pharmacological gout therapy and the establishment of guidelines. His highly cited article describes the 2012 American College of Rheumatology guidelines for managing gout and the treatment and antiinflammatory prophylaxis of AGA (cited484 times) (22). The report gives clinicians with treatment and prevention guidelines for GA. His latest study investigated the effects of wear on plantar pressures and footwear characteristics in people with gout over six months. After six months, the study discovered signs of wear at the worn footwear’s upper, midsole, and outsole. These changes to the structural properties of the footwear may impact forefoot loading patterns in gout patients (23). This latest study illustrates the impact of shoe structural characteristics on gouty patients, who will consider more factors when selecting shoes. It also provides new suggestions for the treatment and prevention of gout.

Notably, eight of the top ten most prolific journals had a high IF. It implies that publishing research on GA in high-quality publications is not a challenge. The Clin Rheumatol, Rheumatology, Arthritis Res Ther, and Annals of the Rheumatic Diseases (ARD) made significant contributions. The following are the reasons why. Firstly, one of the likely explanations for this observation is the IF of these publications. However, we believe that the scientific direction and study topics covered by these journals, more related to scholars’ works, are more likely to encourage them to submit their studies to these journals. To elaborate a little, Clin Rheumatol is a journal of clinical exploration and research in the broad field of rheumatology, focusing on the research for clinical aspects. It includes all current clinical and experimental research trends and the management and assessment of diagnostic and therapeutic approaches for inflammatory, immunologic, and metabolic illnesses. Rheumatology aspires to assist worldwide research and discovery by publishing high-quality scientific publications covering the whole spectrum of rheumatology, from hypothesis-generating fundamental discoveries to translational and clinical research, encompassing adult and paediatric disorders. The Arthritis Res Ther publishes original papers, reviews, comments, and reports on musculoskeletal research and therapy. The journal’s primary focus is on the immunologic mechanisms that contribute to inflammation, injury, and healing in autoimmune rheumatic and musculoskeletal disorders and the translation of this information into breakthroughs in clinical care. Besides, the Annals of the Rheumatic Diseases (ARD) (IF=19.103), in particular, have a higher citation rate and H-index. The ARD is a peer-reviewed international journal that covers all aspects of rheumatology, including musculoskeletal conditions, arthritic disease, and connective tissue disorders. ARD publishes basic, clinical, and translational scientific research, as well as the most crucial management recommendations for a variety of conditions. It belongs to the top Journals in Rheumatology. Furthermore, because these journals are professional periodicals with a high degree of popularity and impact, academics may more readily promote their ideals or opinion in the field of science, allowing them to discuss and exchange their ideas with peers in order to improve their academic level and science ability. Finally, the review cycle of these journals is relatively short. Therefore, articles are more willing to be submitted to them by academics. Based on this tendency, the journals indicated in **Table 5** may continue to be the “primary channel” for future results in this subject. Meanwhile, it also prompted scholars interested in the subject to read these publications more closely.

Among the top 10 most cited articles, research on the treatment of GA accounted for 60% (6/10), indicating that research on the treatment of GA is a hot topic of interest for most scholars (**Table 7**). Three scholars whose articles have been cited more than 450 times are Kottgen, A, Khanna, D. and Schauer, C., respectively. Kottgen, A.’s article from 2013 had the highest cited (491 citations), placing first. His research showed that inhibin-activin signalling pathways and glucose metabolism are implicated in systemic urate regulation. The discovery of new candidate genes for serum urate concentration emphasises the significance of metabolic regulation of urate synthesis and excretion, which may have implications for the treatment and prevention of gout. Hence, it had the highest cited, indicating that other scholars highly acknowledged his work (24). What’s more, Khanna D. et al. present the first American College of Rheumatology (ACR) guidelines for the treatment and antiinflammatory prophylaxis of acute gout attacks in this study (22). His paper offers advice on managing gout, including when to use urate-lowering therapy (ULT), how to treat gout flares, and lifestyle and other medication recommendations. It is beneficial for clinical and scientific researchers as well as patients to gain a better understanding of gout and find official guidelines for the prevention and treatment of gout. In addition, the studies of Schauer C. et a. revealed that in neutrophilic inflammatory models, aggregated neutrophil extracellular traps (AggNETs) reduce inflammation by degrading chemokines and cytokines. On the other hand, the author thinks MSU crystal-activated AggNETs trap and proteolytically degrade neutrophil-derived cytokines and chemokines, thereby reducing inflammation. This mechanism could explain the enigmatic spontaneous remission of acute inflammatory attacks elicited by MSU crystals in people with gout, and it could also be relevant to other types of neutrophilic inflammation. (25).

The timeline view of co-cited references and keywords **(** **Figures 7B**, **8C** **)** revealed the hot spots of common concern all the time are “inflammation,” “nlrp3 inflammasome,” “and canakinumab,” and “ultrasound.” Based on the clustering, we discovered that the majority of studies focused on the basic therapy and diagnosis studies of GA. A critical review (cited 321 times) details the therapy of inflammation by blocking interleukin-1 in humans (26). Similarly, the study conducted by Juliana C. et al. reported that inappropriate NLRP3 activation is involved in the pathogenesis of GA and discovered a new regulatory mechanism that regulates NLRP3 inflammasome activation. (16). Schlesinger, N. et al. showed that Canakinumab significantly relieved pain and inflammation and reduced the risk of a new disease in these AGA patients (27). Ultrasonography has been proven to be a viable and reliable imaging tool for articular cartilage visualisation. According to Filippucci, E. et al., ultrasonography may be useful in determining cartilage involvement in patients with crystal-related arthropathy. (28).

Some emerging research fields are gradually becoming research topics of interest as GA research progresses. **Figures 8A, D** showed the co-occurrence analysis of keywords. The purpose of co-occurrence is to evaluate the link between the items that have been recorded. It is regarded as a helpful tool for anticipating evolution and hot in a specific academic topic. By evaluating the keywords in all of the included publications, we created a network map of the co-occurrence connection. Finally, five possible study directions were identified, which are as follows **(** **Figure 8A** **):** “the inflammatory mechanism of GA,” “the prognosis and epidemiology of GA,” “the research on the diagnosis of GA,” “the clinical research of GA” and “the care and risk factors for GA.” The most common keyword occurrences were “gout,” “hyperuricemia,” “gouty arthritis,” “inflammation,” and “arthritis.” It implies that the majority of the research into GA has concentrated on its causes and mechanisms. Among them, hyperuricemia, which is known to be a risk factor for GA. In the work of Qingxi, Zhang et al. mentioned that hyperuricemia and gout were predicted as the following hot topics (29). Hence, we can conduct more research on hyperuricemia and gouty arthritis in the future. Furthermore, **Figure 8D** shows that the hot words that have been appearing in the past three years are “pathway,” “nf kappa b,” “injury,” “receptor,” and “animal model”. It can be seen from this that in the last three years, people have attached great importance to mechanism research and animal modelling of GA to investigate the treatment mechanism of GA from the standpoint of basic medicine.

An overlay visual graphic comparable to a co-occurrence graph. Items are coloured differently based on the average time of occurrence of the term. It enables immediate tracking of research progress and predicting future hot themes. The varied colours in **Figure 8B** represent the appropriate year of publication. According to the findings, clinical research” accounted for a large portion of blue and green after 2014. It is consistent with the year (2014) when the keyword “randomised controlled trial” in **Figure 8D** began, indicating that after 2014, more studies concentrated on the clinical studies of GA. Nonetheless, “molecular mechanism study” accounted for the majority of the colours green and yellow, implying that after 2018, more research will be focused on the mechanism of GA inflammation. However, A considerable number of nodes of various colours (from purple to yellow) can be observed in each of these five clusters, reflecting a balanced development in each of the five study fields during the previous decade. Furthermore, each direction has seen shifts in research hotspots, demonstrating various advancements.

Based on the bibliometric analysis and visualisation literature, in a way, we can better understand trends and hot spots in GA research. Nevertheless, there are some limitations to this study. Firstly, the data from SCI-expanded were only included for articles and reviews in English. Secondly, because VOSviewer and Citespace cannot analyse the complete text of the publication, certain information may be ignored. Finally, this research has some latency due to some good recently released articles with low Nc being excluded.

## Conclusions

This analysis of bibliometrics shows that the research of GA has a minor fluctuation in the number of articles published over the last decade. However, the overall trend is toward more and more articles being published. The research of GA has a good research prospect. China is the leading producer, and the USA has a more significant influence in this field. The clinical research and molecular biology of GA have attracted extensive attention. The function of molecular pathways of NLRP3, as well as inflammatory Cytokine Levels Regulation, has become a potential hotspot in GA research. It is worth noting that little study has been conducted on the therapeutic effects of Chinese medicine monomers for GA, which should be expanded in this aspect in the future. Our study can be beneficial to scholars to comprehend better the present state of GA research from a macro viewpoint.

## Data Availability Statement

The original contributions presented in the study are included in the article/supplementary material. Further inquiries can be directed to the corresponding author.

## Author Contributions

PD performed the data curation. PD and SW performed the statistical analysis. ZC, YQ, ZM, and JW performed the supervision. PD wrote the original draft of the manuscript. XP, HL, and XS reviewed and edited the manuscript. All authors contributed to the article and approved the submitted version.

## Funding

This work was supported by grants from the Beijing Municipal Administration of Traditional Chinese Medicine 2019 Clinical Collaborative Capacity Construction Project of Chinese and Western Medicine for Major Difficult Diseases.(No. 201803190106). Sub-project name: Staged stepwise treatment of knee osteoarthrosis with Chinese and Western medicine. 

## Conflict of Interest

The authors declare that the research was conducted in the absence of any commercial or financial relationships that could be construed as a potential conflict of interest.

## Publisher’s Note

All claims expressed in this article are solely those of the authors and do not necessarily represent those of their affiliated organizations, or those of the publisher, the editors and the reviewers. Any product that may be evaluated in this article, or claim that may be made by its manufacturer, is not guaranteed or endorsed by the publisher.
